# Improving Electrophoretic Particle Motion Control in Electrophoretic Displays by Eliminating the Fringing Effect via Driving Waveform Design

**DOI:** 10.3390/mi9040143

**Published:** 2018-03-23

**Authors:** Shitao Shen, Yingxin Gong, Mingliang Jin, Zhibin Yan, Chang Xu, Zichuan Yi, Guofu Zhou, Lingling Shui

**Affiliations:** 1 National Center for International Research on Green Optoelectronics, South China Normal University, Guangzhou 510006, China; shenshitao@m.scnu.edu.cn (S.S.); yxgong-aoe@m.scnu.edu.cn (Y.G.); jinml@scnu.edu.cn (M.J.); zhibin.yan@m.scnu.edu.cn (Z.Y.); xuchang@m.scnu.edu.cn (C.X.); guofu.zhou@m.scnu.edu.cn (G.Z.); 2 Guangdong Provincial Key Laboratory of Optical Information Materials and Technology and Institute of Electronic Paper Displays, South China Academy of Advanced Optoelectronics, South China Normal University, Guangzhou 510006, China; 3 Zhongshan Institute, University of Electronic Science and Technology of China, Zhongshan 528402, China

**Keywords:** electrophoretic particle, fringing field, driving waveform, voltage signal

## Abstract

Electrophoretic display is realized by controlling colored nanoparticles moving in micrometer spaces via electrophoresis. The quality of information display is therefore affected by the unsynchronized particle moving speed and the mismatched electric signal according to the crosstalk of the electric field and inhomogeneous material distribution. In this work, we analyzed the mechanism of a fringe phenomenon that affected the information display quality of electrophoretic displays (EPDs). Electrical driving waveforms (voltage signals) are designed to reduce the fringe phenomenon. By using the optimizing driving waveform, we proposed that the fringe phenomenon is quantified as gray value that can be diminished by 25.5, while keeping a response time of 200 ms.

## 1. Introduction

Electrophoretic displays have been the subject of intense research for many years because of their wide market potential [1,2]. Among electronic displays, electrophoretic displays have the advantages of bistability for low consumption usage, light reflective characteristics and a wide view angle for comfortable readability, and wetting process for flexible displays [3]. Recently, electrophoretic displays (EPDs) have been widely used in the fields of electronic books, electronic labels and smart watches [4]. However, the schemes used to drive EPDs to achieve multilevel gray scales are still unsatisfactory, especially for displaying good quality information at high speeds. Therefore, there are limits regarding their applications for webpage browsing and video playing. In addition, it is difficult to guarantee the display quality when using conventional short-term driving waveforms to play videos on commercial EPDs. Thus, the optimization of display quality during the short driving process is still urgently required [5].

In an electronic display device, in general, an active matrix backplane is used to scan and realize a driver on the front displaying materials such as electrophoretic particles. Thin film transistor (TFT) functions as a switch is to scan line-by-line for each row and applies independent signals to each column; thus, TFT can be used for high throughput electrode array control [6,7]. The function of gate lines is to control whether the voltage is applied on pixel electrodes in one row, and the source electrode connects to the pixel electrodes. When a voltage is applied to the source electrode, the electric field is generated between the common electrode and the pixel electrode, driving the charged particles to move. By controlling the time and space sequence of voltage pulses, the electrophoretic particle motion could be controlled. The shape and form of the electric signal here is called the driving waveform. This plays a key role in controlling the image quality of a display device [8,9].

### 1.1. Mechanism of Electrophoretic Displays (EPDs)

**Figure 1:**
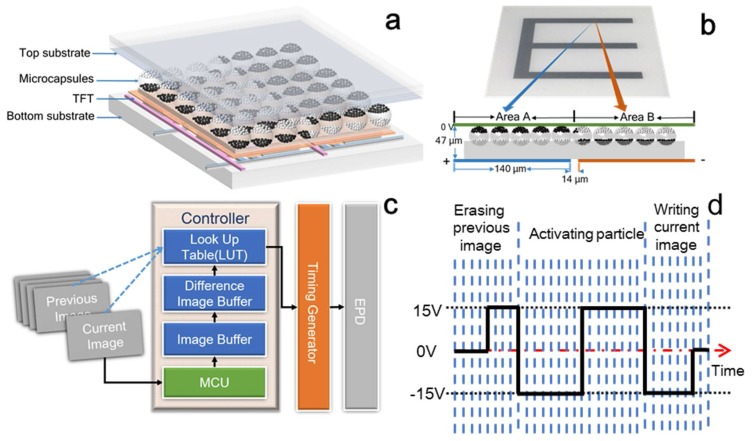
Structure, principle, operational modes and driving scheme of electrophoretic displays (EPDs). (**a**) Schematic drawing of the EPD structure, from bottom to top is the bottom substrate (glass) → thin film transistor (TFT) (pixel electrode) → microcapsule (white and black electrophoretic particle) → common electrode → cover plate (including optical film and water proof protection); (**b**) The cross-sectional view of the EPD device (bottom), showing the electrophoretic particle distribution in the microcapsules corresponding to the white and black areas on the real sample image (top); (**c**) Operational modes in the EPD controller; (**d**) A typical EPD driving waveform containing three phases for image updating.

Figure 1a shows the schematic of a microcapsule-based EPD device. The black and white particles encapsulated in the microcapsules are positively and negatively charged, respectively. The microcapsules are sandwiched between the common electrode and pixel electrodes. The transparent conductive film covering all pixels is used as the common electrode. The pixel electrodes can provide alternative voltages respectively to actuate white and black particles to move in the microcapsules along the direction of electric field. In order to achieve accurate graytones, the applied voltage on the common electrode can be adjusted from −3 V to 0 V. Therefore, the obtained image quality like gray scale or contrast ration is closely related to the applied voltage on EPDs. 

Particles with positive or negative charges would be motivated along the field direction in a different way when changing the applied voltage, and then indirectly change the electric field distribution.

In general, the materials in the microcapsule include insulating oil, charge control agents, electrophoretic particles and density balance agents [1,10]. In particular, the parameters relative to electrophoretic phenomenon are the mass and zeta potential of the particles, the dielectric characteristics and viscosity of the insulating oil, and the electric field strength. The dependence of the electrophoretic driven particle moving has been studied based on the hydromechanics theory, as described in Equation (1) [11].

(1) $$v_{i}=\frac{qE_{i}}{b_{i}}\left(1\pm e^{-\frac{b_{i}}{m}t}\right)$$

where *q* is the electrical charge of the particle, *m* is the mass of the particle, and *b*_i_ is the fluid viscosity. The velocity (*v*_i_) is proportional to the electric field strength between the pixel and common electrodes (*E*_i_). There is a negative correlation between *v*_i_ and *b*_i_. In the same microcapsule, *b*_i_, *m* and *q* are considered as constants; therefore, the *E*_i_ generated by electric potential has a significant effect on *v*_i_ which relates directly to the display process as the key influence factor for an EPD. To address the fringe phenomenon, the focus area is the two adjacent pixel units located on the boundary line between white and black regions, designated as Areas A and B in Figure 1b. The incident light from the environment is partially absorbed with the rest light reflected back to the top plate to the reader’s eyes. 

### 1.2. Electrical Driving Scheme for EPDs

As a voltage-controlled device, the electrical waveform is the key parameter which directly relates the movement control of charged particles to suitable driving speed, and image quality and contrast. A typical waveform mode in the EPD controller is illustrated in Figure 1c. The driving waveforms are written into a lookup table (LUT) for better selection, by considering both previous and current images [12]. The direct direct current (DC) drive would cause non-identical distribution of particles for different display processes, inducing ghost images. The particle distribution shows a nonlinear relationship with voltage and the characteristic of response latency [13]. Therefore, the most widely used driving waveform is composed of three phases of erasing the previous image, activating charged particles, and writing the current goal image to achieve accurate gray levels, as demonstrated in Figure 1d [14]. 

The rail-stabilized driving scheme has been found to display accurate graytones and eliminate ghost images on EPDs. In a rail-stabilized driving scheme, the “rail” optical state is one of the two gray levels: black and white, and it will first be displayed to reset the display in a unite optical state. The distribution of particles will reach a limit state, since white and black particles will reach the top and bottom in a microcapsule, respectively. On the basis of this state, accurate goal graytones can be achieved. By resetting to “rail” optical state, the ghost image problem can be solved on EPDs. However, the display switches between black and white states will take a long time and bring visual flickers [15]. The characteristics of the response latency under different image retention time were discovered, then a compensated driving waveform design based on the analysis of the EPDs electro-optical responses under different image retention time was proposed [13]. This waveform can reduce the number of states and maintain a high display quality on EPDs. A multilevel driving waveform for reducing the response time on EPDs has been used [16]. However, once 20 V and 5 V are applied as two additional voltages, the driving system becomes more complicated. Schemes with an activation pattern have been proposed to reduce the influence of the ghost image during the driving process [17]. However, this scheme is not helpful to improve the response speed of EPDs. Kao et al. [18] proposed an entire system with a new driving waveform aiming at realizing applications of EPDs with video-like display property. A fringe effect on EPDs was reduced by using the new driving waveform. However, it was difficult to completely clear the fringe effect under the playing speed of the video; thus, an effective driving scheme for eliminating the fringe effect on EPDs is still required.

A common electrode design method named finger-on-plane (FOP) has been proposed to eliminate fringe field effects for liquid crystal on silicon (LOCS) [19]. For facilitating the rotation of liquid crystal molecules in one direction when appropriate voltages are applied to the pixels, the common electrodes are designed to tilt slightly away from the vertical lines. A circularly polarized light illuminated reflection-type LOCS device was investigated to eliminate the fringing field effect of the vertically aligned cell compared with the linearly polarized light [20]. Simulated results of the fringing field effects on reflective LOCS were discussed and verified. When the adjacent pixels were operated at different voltages, the lateral component of the fringing field can degrade the electro-optic performance of the device. The fringing field of liquid-crystal displays causes a crosstalk between neighboring pixels and indirectly leads to the blurring effect which influences the diffraction efficiency of holograms. Moreover, in both simulation and experimental measurements of subpixel Jones matrices, the diffraction efficiency increases with gray value difference in the hologram, with vertical gratings showing better efficiency than horizontal gratings [21]. The cost will increase when changing the device structures. Therefore, the method based on driving waveform optimization shows high potential in applications. 

In this work, we first identify the refreshing process and corresponding waveforms causing the fringe phenomenon in electrophoretic displays. The generation mechanism of the fringe phenomenon was analyzed by simulating the electric field distribution in two adjacent square pixels with the length of 140 μm. Experiments were carried out to investigate the effect of diffusion and transverse fields induced by 15 V and 30 V potential differences. Results from experimental measurements and simulation are consistent. Improved driving waveform design was validated in a commercial EPD, showing obvious improvement in image displaying without a fringing effect. As a result, the image ghost contours were reduced by 25.5 gray value difference in 160 ms without changing any hardware of the EPD device such as structure and materials.

## 2. Experimental

### 2.1. Devices and Software

Commercial E-ink electronic-paper displays Ed060SC7(lf)c1 (E ink Holding Inc., Hsinchu, Taiwan) was used for the experiments in this work. Three voltage levels are available on the pixel electrodes: +15 V, −15 V, and 0 V, and the range of voltage applied on the common electrode is from −3 V to 0 V. Frame time is 20 ms, frame rate is 50 Hz. A four-level gray scale driving scheme was employed in the experiment. W, LG, DG, and B represent white, light gray, dark gray, and black of the four levels of gray scale, respectively. The symbol of W-B represents the driving process from white to black.

The integrated driving system EPD04A (iRex Technologies, Eindhoven, The Netherlands) with light sources, a digital video camera Matnta G201B ASG (ALLIED Vision Technologies, Stadtroda, Germany), a computer with waveform editor and driver and detector proprietary software were used. The driving waveform editor was called Project_epaper_2301 (iRex Technologies, Eindhoven, The Netherlands). It offers unit time adjustment, voltage selecting, and waveform design and loading, etc. As a driver and detector, the software named WFD_nex (iRex Technologies, Eindhoven, The Netherlands) provides image loading, driving waveform selecting, video camera information collecting and reflectance conversing.

DC Power supply device Agilent E3631A (Agilent Technologies, Santa Clara, CA, USA) was used to continuously applying voltages on a printed circuit board (PCB) which was connected to the EPD and controlled by the integrated driving system EPD04A. Waveform data edited by software in computer, was then transferred to PCB and transformed into voltage sequences. In the end, the PCB provided the designed waveform to drive EPDs to display information. 

A commercial finite element simulation software COMSOL Multiphysics 5.3 (COMSOL Inc., Stockholm, Sweden) was used to conduct the electric field simulation analysis. Images were analyzed using Matlab to obtain standardized gray values and contrast ratio.

### 2.2. Characterization

A stereomicroscope SZ760T2LED (Optec optical instrument Co., Ltd., Chongqing, China) with a charge-coupled device (CCD) connected to a computer, was used to capture images when studying the fringe phenomenon. The parameters for capturing images were set as exposure rate of 5000, contrast ratio of 32, and the R, G, B color of 793, 500 and 520, respectively.

## 3. Results and Discussion

### 3.1. Process of the Fringe Phenomenon on EPDs

The fringe phenomenon typically occurs as a boundary line between two different graytones, with distinguishing gray difference compared to the gray level of the left and right sides. Thus, it is typically viewed as contour lines of ghost images. When the value of gray difference is positive, a white line can be observed around the edges; and correspondingly, a black line occurs at a negative gray difference. When a driving waveform is applied, this phenomenon is commonly observed by a short driving process with a straightforward driving scheme which has also been reported by Kao et al. [18]. 

**Figure 2:**
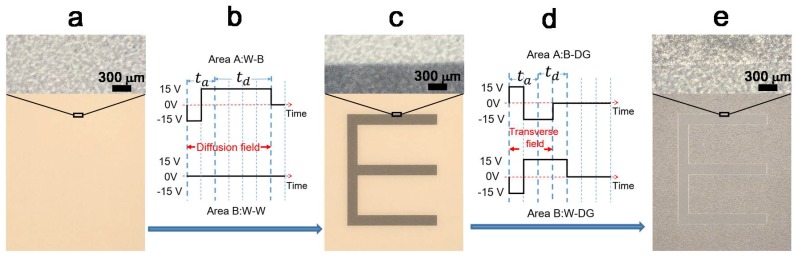
Process of the fringe phenomenon. (**a**) The original white background of an EPD screen (12.4 cm × 9.3 cm); (**b**) Driving waveform used for the first processing from white background to the black “E”. Areas A and B are the updating waveforms corresponding to W-B and W-W, respectively. *t*_a_ and *t*_d_ represent the activating particle phase and straightforward driving phase, respectively. The minimum fixed time for a voltage continuity is 20 ms. In this waveform, the diffusion field lasted for 120 ms when the 15 V voltage difference was produced between Areas A and B; (**c**) A black “E” letter appears after the first refreshing process driving by the waveform in (**b**); (**d**) Driving waveform used in the second refreshing process from the black “E” to the dark gray (DG). Areas A and B were the updating waveforms corresponding to B-DG and W-DG, respectively. The transverse field lasted for 60 ms with 30 V voltage difference applied between Areas A and B; (**e**) A dark gray image of the “E” letter by the second refreshing process driving by the waveform in (**d**). The average gray value of the background, contour and “E” was calculated using Matlab with the gray value being set from 0 to 255.

In this work, the fringe phenomena were observed at various waveforms. A typical generating process of the fringe phenomena is illustrated in Figure 2. The flow process from Figure 2a–c represents a refreshing from a white background to an absolute black “E”, with Figure 2b showing the corresponding driving waveform. Ideally, the refreshing process from Figure 2c–e represents the image changes from the black “E” to a dark gray (DG) of the entire screen by using the waveform in Figure 2d. However, a white contour is generated at the edges of “E” where the previous white background and the black “E” are located. This fringe phenomenon worsens the ghost image problem of EPDs.

At the first stage, from the original white background to the black “E”, Areas A and B were designed to drive electrophoretic particles to show W-B and W-W, respectively. Within 120 ms duration, the black Area A and white Area B appeared on the EPD screen. The diffusion field here is defined as shown in Figure 2b, representing the electric field generated in Area A diffusing toward the region in Area B. By applying the second stage waveform (Figure 2d), the change of Area A from B to DG and Area B from W to DG were obtained (Figure 2e). When a 30 V voltage difference was produced between Areas A and B, within 60 ms continuous driving time, the transverse field was generated (Figure 2d). Substantial horizontal components were generated because of the −15 V voltage on Area A and the +15 V voltage on Area B. 

The cause of the fringe phenomenon is then analyzed and discussed as follows. In our experiments, we discovered that the fringe phenomenon appeared at the border of two different graytones such as the ghost image of the letter “E” with the gray value difference of 25.5 between contour and adjacent areas, as shown in Figure 2e. Such a fringe phenomenon is significantly different from typical crosstalk which happens as undesired vertical or horizontal communication due to parasitical capacitances between the source line and the pixel electrode or the common electrode. In fact, the fringe phenomenon that resulted from the interference between adjacent pixels will be affected by the fringing field. Moreover, once the horizontal component of the fringing field drives charged particles to move in a horizontal direction, it will be difficult to restore the particle distribution by its own vertical driving electric field. A fringe phenomenon then remains on the screen for subsequent frames, reducing the display quality. Thus, deeply understanding the fringe phenomenon and diminishing it by waveform design are very important for the electrophoretic particle manipulation in EPDs. 

The pixel electrode and common electrode in a pixel of EPD is equivalent to a parallel plate capacitance. Charges will aggregate on the surface of a rectangle pixel electrode with higher charge density at the four corners [22]. The charge density at the node (*x*, *y*, *z*) of a pixel electrode is calculated as Equation (2):

(2) $$\sigma \left(x,y,z\right)=\frac{2\pi \varepsilon V_{0}}{\oint \frac{dS_{1}\;\left(x^{\prime },y^{\prime },z^{\prime }\right)}{R_{1}}-\oint \frac{dS_{2}\;\left(x^{\prime \prime },y^{\prime \prime },z^{\prime \prime }\right)}{R_{2}}}$$

with *M* (*x*, *y*, *z*), a node on arbitrary plate of capacitance; *dS*_1_ (*x*′, *y*′, *z*′) and *dS*_2_ (*x*″, *y*″, *z*″), the area element on the upper plate and lower plate, respectively; *R*_1_, the distance between *M* and the node (*x*′, *y*′, *z*′); and *R*_2_, the distance between *M* and the node (*x*″, *y*″, *z*″). *ε* is the permittivity for the capacitance. *V*_0_ is the potential difference between two plates. *σ* (*x*, *y*, *z*) is the charge density of the plate.

As the surface charge distribution is inhomogeneous on the plate, the electric field around the plate’s boundary extends to the exterior area and therefore, generates the fringing field. Such an obvious effect cannot be neglected when analyzing the relationship between applied voltages on plates and the movement of particles. 

**Figure 3:**
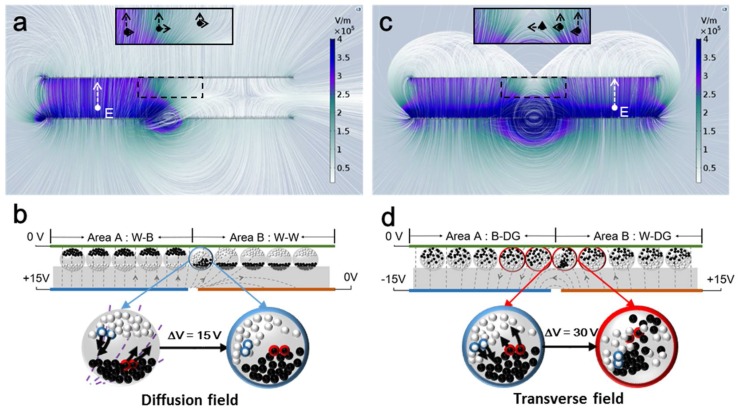
Electric field and particle distribution of two adjacent pixels. (**a**) Distribution of the diffusion field in Areas A and B. The voltage of +15 V and 0 V was applied to the pixel electrode of Areas A and B, respectively, generating a 15 V voltage difference; (**b**) Schematic diagram of the particle distribution in the action of diffusion field effect, the strongly affected microcapsules are marked in blue; (**c**) Distribution of the transverse field in Areas A and B. A voltage difference generated between two bottom electrodes of Areas A and B was 30 V by applying −15 V and +15 V voltage on the pixel electrodes of Areas A and B, respectively; (**d**) Schematic diagram of the particle distribution in the action of the transverse field effect. Microcapsules that are strongly affected by the transverse field are marked in red.

To understand the relationship between the fringing field and voltage applied on a pixel electrode, the electric field between two pixel areas was simulated using COMSOL Multiphysics 5.3. As shown in Figure 3, the horizontal component of the fringing field is found between two pixel areas, and the interior of the pixels as well. Therefore, the edge line is in fact widened. In the simulation, the side length of pixel electrode was set to 140 μm, the applied voltage on common electrode was 0 V. The distance between the common electrode and pixel electrode (cell gap) was 47 μm. The length and width of the common electrode were 294 μm and 140 μm, respectively. The inter-pixel gap between Areas A and B was 14 μm. The set of Maxwell equation is formulated as:

(3) $$\{\begin{array}{c} E=-\nabla V\\ \nabla \cdot E=\frac{\rho_{v}}{\varepsilon_{0}\varepsilon_{r}} \end{array}$$

where *E* is the electric field, *V* is the potential, *ρ*_v_ is the volumetric free charge density in the electric plates. *ε*_r_ is the permittivity. The generated diffusion field is shown in Figure 3a when the applied voltage on the pixel electrodes of Areas A and B are 15 V and 0 V, respectively. The magnified area was marked with black outline shows that diffusion field strength was reduced from approximately 3.5 × 10^5^ V/m to 0.5 × 10^5^ V/m. The vertical component of the diffusion field near to the inter-pixel gap and upper plate gradually diminished from Area A to Area B. The horizontal component remained in this magnified area. Corresponding particle distribution is shown in Figure 3b under the effect of the diffusion field. It is clearly seen that the microcapsules in Area B and near the gap was significantly affected by the diffusion field. Particles distributed in the microcapsule marked as blue moved both in vertical and horizontal directions in the diffusion field. It should be noted that, at this stage, the fringe phenomenon might not appear because a large number of white particles in blue microcapsule are still on the upper surface with little difference compared to the white background.

When a 30 V voltage difference in the horizontal direction was applied as the designed waveform with −15 V and +15 V on pixel electrodes of Areas A and B, respectively. An obvious fringe phenomenon was observed, as shown in Figure 3c, and the corresponding particle distribution is presented in Figure 3d. The microcapsules near the gap are significantly affected by the transverse field, marked as four red microcapsules. In this situation, the fringe phenomenon is wider than the common electrode gap, and becomes more obvious. In this analysis, the focus area is from the common electrode to the position of 19 μm below, containing most microcapsules in an EPD device, called effective area. It is certain that the electric intensity of both diffusion and transverse fields in this effective area is less than the average electric intensity in the pixel area. The horizontal component of the fringing field in the effective area can change particle distribution which might be difficult to restore by a general vertical driving scheme, and thus, would remain for the long term. The vertical component of the fringing field in the effective area is insufficient to drive particles to reach the goal gray level. Therefore, in the process from W to DG, the fringe phenomenon will be demonstrated as a white line lying on the DG background. 

### 3.2. Diminish Fringe Phenomenon by Optimizing Driving Waveform 

From the simulated results as shown above, it is clear that once the transverse field is generated, it will induce undesirable particle distribution in the horizontal direction and weaker particle movement in the vertical direction. Both effects are detrimental to the realization of target gray levels. These make the fringe phenomenon even more severe than that from the diffusion field. Therefore, to eliminate the influence of the fringing field, we can highly improve the EPD displaying performance. 

**Figure 4:**
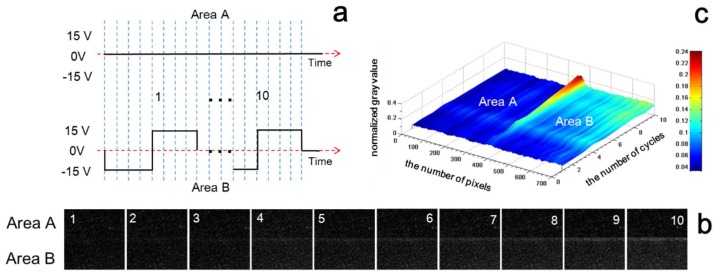

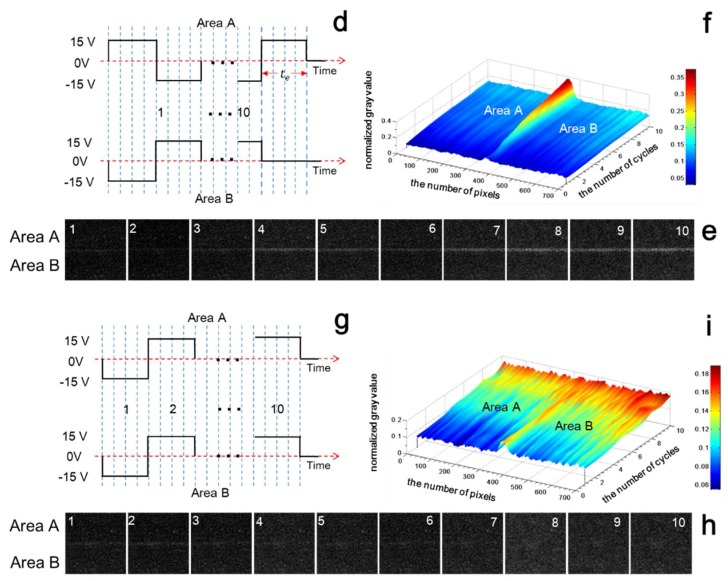
Investigation of the diffusion and transverse field effects on fringe phenomenon. (**a**) The driving scheme for finding out the relationship between diffusion field and fringe phenomenon. The unit time of waveform is 20 ms, and the initial gray scale is black; (**b**) Corresponding pictures at the end of each cycle of the waveform in (**a**); (**c**) 3D diagram based on numerical analysis of the 10 images in (**b**); (**d**) The driving scheme for finding out the relationship between the transverse field and fringe phenomenon. The unit time of the waveform is 20 ms, and the initial gray scale is black; (**e**) Corresponding pictures at the end of each cycle of the waveform in (**d**); (**f**) 3D diagram based on numerical analysis of the 10 images in (**e**); (**g**) The optimizing driving scheme to avoid both transverse and diffusion field effects on fringe phenomenon. The unit time of the waveform is 20 ms, and the initial gray scale is black; (**h**) Corresponding pictures at the end of each cycle of the waveform in (**g**); (**i**) 3D diagram based on numerical analysis of the 10 images in (**h**).

In this work, we propose and verify a method to diminish fringe phenomena via optimizing the electric driving waveforms. As shown in Figure 4, three experiments were carried out to investigate the influence degree of diffusion and transverse fields. The real images driven at different driving stages were captured using a CCD camera equipped on a stereomicroscope.

First, the driving scheme in Figure 4a was used to find out the relationship between the diffusion field and fringe phenomenon by only applying ±15 V square waveform with 0.5 duty cycle and 160 ms periods on pixel electrodes of Area A, keeping pixel electrodes of Area B at 0 V. Figure 4b shows the captured displayed pictures by applying the waveform ten times. Each picture was taken at the end of each waveform cycle. The gray scale transformation and gray value normalization were obtained using Matlab software. The picture in Figure 4c was obtained by averaging the 10 pictures of Figure 4b. The 10 columns correspond to 10 repetitive cycles, and 700 pixels were obtained from the captured images. The gray difference in this picture is larger than the initial state over time.

Secondly, the experimental driving scheme of the transverse field was carried out in a similar way. Square waveforms of ±15 V with 0.5 duty cycle and 160 ms periods were applied on the pixel electrodes of Areas A and B, respectively; however, the directions of the applied voltages were opposite (Figure 4d). Here, *t*_e_ is the phase at the end of the designed waveforms, which is applied to ensure that both Areas A and B turn black at each driving cycle. In this way, the edge effect can be viewed clearly. Without the *t*_e_ phase, during the driving scheme of the transverse field, when Area B turns black, Area A turns white. With the application of the *t*_e_ phase, both Areas A and B can be driven to the similar states of black. Figure 4e,f present the obvious fringe phenomena resulting from the transverse field driving process. Comparing Figure 4c–f, it is obvious that the transverse field is more effective than diffusion field considering the generation of fringe phenomenon. Therefore, high voltage difference between adjacent pixels should be avoided to diminish the transverse field generation. 

Accordingly, a method to reduce the influence of both transverse and diffusion fields was proposed and verified. The activating scheme was designed based on synchronously driving particles in adjacent pixels. The square waveform with 0.5 duty cycle and 160 ms periods was synchronously applied to the pixel electrodes of Areas A and B (Figure 4g). The obtained results are shown in Figure 4h,i. The initial image for this experiment was produced by repeating the diffusion field driving scheme 10 times. After four activating cycles, the fringe phenomenon was obviously lower; and the line induced by the fringe phenomenon completely disappeared after 10 cycles. 

These results show that the fringe phenomenon can be clearly reduced by optimizing the driving waveforms. Conventionally, when +15 and −15 V voltages are applied on the pixel electrodes of Areas A and B, respectively, the gray scales are refreshed toward the opposite direction. However, an obvious transverse field would appear and induce obvious fringe phenomena. In our strategy, 0 V voltage is applied to Area B to generate a 15 V voltage difference to avoid the transverse field. In addition, in order to reduce the effect of the diffusion field, the activating driving scheme shown in Figure 4g was designed to achieve the improved image quality in Figure 4h. In EPD devices, the activating driving scheme is designed with the same voltage sequence of each waveform so that the particles near the line edges are remixed well. These particles are usually affected by an uneven electric field in the vertical direction near to the line edges. The activating scheme for remixing particles in vertical direction can reduce the difference of gray values in adjacent areas. Meanwhile, the synchronized motion of particles can reduce the effect of horizontal electric field in adjacent areas, thereby reducing the fringe phenomenon.

**Figure 5:**
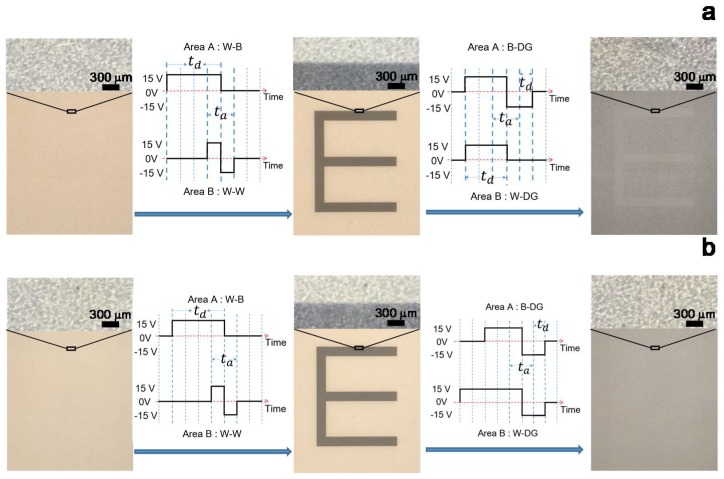
Driving waveform optimization. (**a**) The optimized driving waveform by reducing transverse and diffusion field effects. (**b**) Integrated waveforms with functions of reducing fringing effect and gray scale homogenization. In (**a**,**b**), from left to right are the initial state of the EPD device → driving waveforms for display a black “E” → the black “E” on the screen → driving waveform to erase the black “E” to DG → the resulting DG quality.

In order to verify the mechanism by which optimization of the driving waveform can improve the displaying quality of real images, a driving scheme was applied to the display and a black “E” was erased on an EPD device. The optimized driving waveform is shown in Figure 5a. This driving waveform was verified as the same refreshing process as shown in Figure 2. To achieve the goal gray levels, the straightforward driving phase is denoted as *t*_d_. The activating driving phase *t*_a_ aims to weaken the interference of the diffusion field, 40 ms was selected to be twice of the unit time period of waveforms (20 ms) without scarifying the straightforward driving time *t*_d_. To avoid producing the transverse field, the applied voltage is positive and negative before and after *t*_a_ phase, respectively. Therefore, *t*_a_ phase is in the middle stage of the optimized waveform rather than at the beginning of the waveform, which is shown in Figure 2. At *t*_a_ stage, the applied voltage needs to be in a sequence from positive to negative from the front to the back. Comparing with the *t*_d_ phase in Figure 2d, the *t*_d_ phase of the waveform for B-DG shown in Figure 5a has been delayed for 40 ms to avoid high voltage difference in the *t*_d_ phase of W-DG. We have tried various waveforms with fixed driving time of 160 ms. The result with *t*_a_ = 40 ms, *t*_d_(+) = 80 ms, *t*_d_(−) = 0 ms demonstrates the best displaying performance in diminishing the fringing effect in the W-B and W-W refreshing processes. The longest driving time of this driving waveform is 160 ms because the realization of the longest refreshing process of W-B (+15 V) and B-W (−15 V) will cost 80 ms, respectively. In order to reduce the driving time and keep the gray value constant, the time integral of voltage was kept constant with +15 V for 80 ms in Figure 5a (W-B), and −15 V for 20 ms and +15 V for 100 ms in Figure 2b (W-B), respectively. In general, the voltage at the end of the waveform contributes more to the gray value. Thus, +15 V for 40 ms was added in the front of the waveform for B-DG (Figure 5a) to offset *t*_a_ in Figure 2b (W-B) to obtain the waveform for B-DG in Figure 5a. 

The initial idea for designing waveforms for a bistable property is simply applying zero voltage when pixels remain at the same state like the pixels in Area B of Figure 2b and Figure 5a. The waveform in Figure 2b can remain the W-W state with 0 voltage. However, such a scheme will make the next step activation more difficult according to the inactivated particles. Therefore, the *t*_a_ phase is added to the waveform in Figure 5a for W-W. By using this optimizing driving waveform, the image ghost contours can be completely erased without changing the EPD structure or materials for the same period of driving time. Comparing Figure 5a to Figure 2e, the gray value difference was clearly reduced from 25.5 to ~0 (undetectable).

In Figure 5a, the letter “E” is still distinguishable from the DG background according to its low evenness of gray scale. To further improve the image quality, a combined driving scheme aiming at homogenizing the gray scale was added, as shown in Figure 5b. It was found experimentally that the uniformity of particle distribution obtained by using the refreshing process from black toward gray was better than that from white toward gray. The mechanism for this has not yet been fully understood. The waveforms for W-DG in Figure 5a,b are significantly different for optimizing the low evenness of gray scale. The waveform in Figure 5a drives the pixel from white to target DG directly. In our experiments, we found that the black particles move quickly to form ring structures in the microcapsules when driven directly from white to DG. Therefore, the obtained DG stage is not very uniform. In Figure 5b, the waveform drives the pixel at two stages, from white to black and from black to target DG. Therefore, better uniformity could be achieved. Such a gray scale homogenizing driving scheme is well integrated with eliminating fringe phenomenon scheme. The kernel of the homogenizing scheme is to improve the uniformity of the white and black particle distribution near the upper plate. It costs 100 ms to obtain a uniform dark situation as an initial state. Accordingly, the longest driving time (or refreshing time) was 200 ms, considering each W→B and B→W refreshing processes costs 100 ms. As seen from Figure 5b, the ghost image is completely eliminated by this optimized driving waveform. 

## 4. Conclusions

An optimized short-term driving waveform to reduce the fringe phenomenon has been presented and verified in this paper. It was confirmed that the refreshing processes and corresponding waveforms are the main reasons behind the fringe phenomenon. The mechanism of the fringe phenomenon was analyzed by simulating the diffusion and transverse fields. The undesired particle distributions in these two kinds of fringing fields were noted to correlate with the fringe phenomenon. Experiments were carried out to investigate the influence degree of the diffusion and transverse fields, and establish the corresponding relationship between the fringe phenomenon and the fringing fields. The proposed driving waveforms have been validated in a commercial EPD device. With the driving time limited to 200 ms, the fringe phenomenon was quantified as the gray value difference which could be reduced by 25.5. Therefore, this work could be applied for improving electrophoretic particle motion control, and especially for high quality information displaying of EPDs.
